# Rapid reinfection with SARS-CoV-2 variant-of-concern Alpha detected in a nurse during an outbreak at a non-covid inpatient ward: lessons learned

**DOI:** 10.1186/s13756-021-01008-4

**Published:** 2021-09-26

**Authors:** Jelle Koopsen, Mireille Dekker, Philip Thung, Marcel Jonges, Harry Vennema, Tjalling Leenstra, Dirk Eggink, Matthijs R. A. Welkers, Peter A. A. Struijs, Chantal Reusken, Rosa van Mansfeld, Menno D. de Jong, Janke Schinkel, Ingrid J. B. Spijkerman

**Affiliations:** 1 Department of Medical Microbiology and Infection Prevention, Amsterdam Infection and Immunity Institute, Amsterdam UMC, University of Amsterdam, Amsterdam, The Netherlands; 2 Department of Medical Microbiology and Infection Prevention, Amsterdam UMC, Vrije Universiteit Amsterdam, De Boelelaan 1118, 1081 HV Amsterdam, The Netherlands; 3 Department of Occupational Health and Safety, Amsterdam UMC, Amsterdam, The Netherlands; 4 Centre for Infectious Disease Control, WHO COVID-19 Reference Laboratory, National Institute for Public Health and the Environment (RIVM), Bilthoven, The Netherlands; 5 Department of Infectious Diseases, Public Health Service of Amsterdam, Amsterdam, The Netherlands; 6 Department of Orthopedic Surgery, Amsterdam UMC, University of Amsterdam, Amsterdam, The Netherlands

**Keywords:** Nosocomial transmission, Outbreak investigation, Infection prevention and control, Infection control guidelines

## Abstract

We describe the lessons learned during a SARS-CoV-2 variant-of-concern Alpha outbreak investigation at a normal care unit in a university hospital in Amsterdam in December 2020. The outbreak consisted of nine nurses and two roomed-in patient family members. (attack rate 18%). One nurse tested positive with a phylogenetically distinct variant, after a documented infection 83 days prior. Three key points were taken from this investigation. First, it was controlled by adherence to existing guidelines, despite increased transmissibility of the variant. Second, viral sequencing can inform transmission cluster inference, but the epidemiological context is essential to draw appropriate conclusions. Third, reinfections with Alpha variants can occur rapidly after primary infection.

## Introduction

After the initial detection of severe acute respiratory syndrome coronavirus 2 (SARS-CoV-2) Variant-of-Concern Alpha (PANGO lineage B1.1.7) in the United Kingdom during late autumn 2020 it spread rapidly and was detected in the Netherlands soon after [1]. Healthcare workers (HCWs) are at an increased risk of being exposed to SARS-CoV-2 but also of being a source of transmission [2]. The Alpha variant has an increased transmissibility compared to the predecessor lineages [3]. Such changing characteristics require continuous reassessment of infection prevention and control practices, but also of the interpretation of outbreak investigations. Here we describe the lessons learned on outbreak investigation and containment from an Alpha variant outbreak at the Amsterdam University Medical Centers.

## Epidemiological context of the outbreak

Nine nurses and two roomed-in patient family members tested PCR positive for SARS-CoV-2 in December 2020 in an orthopedic ward at a hospital in the Netherlands that could accommodate 20 patients. All patients were questioned for COVID19 related symptoms upon admission. Based on their answers, patients with clinical symptoms were placed in isolation and tested for SARS-CoV-2. When tested positive, patients were admitted to the COVID19 cohort ward. None of the admitted patients developed symptoms during their admission.

The index nurse, case number 001, tested positive following an earlier confirmed SARS-CoV-2 infection of a household contact. She developed symptoms two days later. The outbreak within the ward was recognized a day later, when nurse 002 tested positive. This nurse had been in contact during working hours and during outdoor leisure activities with nurse 001 who, at that time, did not have any SARS-CoV-2 related symptoms.

**Fig. 1:**
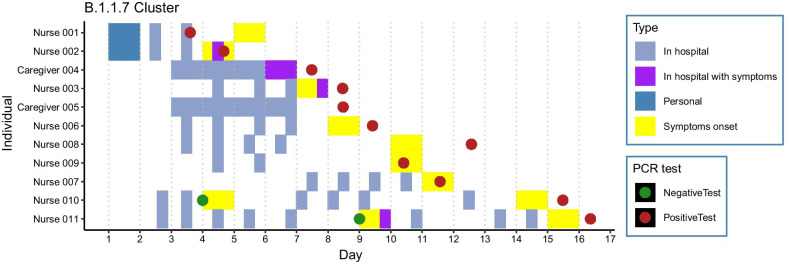
Shifts at the ward from nurses and roomed-in patient family members. Outbreak day 1 was defined as the day of personal contact between nurse 1 and nurse 2

The remaining seven nurses and two roomed-in patient family members developed symptoms and tested positive in the following eleven days (Fig. 1).

The roomed-in patient family members, case number 004 and 005, were members of one household and took care of their admitted family member in close collaboration with the ward personnel. Rooming-in was ended directly after the positive test of informal caregiver 004. Strikingly, the patient did not develop symptoms and repeatedly tested negative. The patient remained SARS-CoV-2 negative, despite repetitive testing.

Nurse 007 tested positive on day eight of the outbreak, after a documented SARS-CoV-2 infection 83 days prior. Nurse 007 reported headache and a slightly elevated temperature in contrast to more severe symptoms during the first infection when fever, dyspnea, respiratory distress and anosmia were present. Nurse 010 and nurse 011, tested negative one week before testing positive. Both nurses reported a sore throat as reason for the initial testing. For nurse 010, fever, muscle strain, and nausea were the reason for the second test. Nurse 011 was retested due to a persisting sore throat, the development of a cough, and a hoarse voice. All cases were followed-up by the department of Occupational Health & Safety. No hospitalizations were reported and at the end of follow-up all nurses fully recovered.

## Containment of the outbreak

To mitigate the outbreak, the importance of the prevailing infection control measures was stressed. The measures included social distancing, capacity limits for personnel break rooms and changing rooms, universal masking with type IIR surgical masks in case a distance of 1.5 m could not be guaranteed, restricted access to the hospital for visitors (one visitor per patient, per day) and PCR testing and domestic quarantine of employees with symptoms in addition to standard precautions and isolation precautions. Infection control practitioners visited the ward each working day during the outbreak period to advise and observe practice.

SARS-CoV-2-positive nurses were put under domestic quarantine at least 7 days after onset of symptoms and until symptom-free for more than 24 h according to national guidelines [4].

For each case, contacts in the work setting were traced. Interestingly, no high-risk contacts (> 15 min at < 1.5 m without a mask) were reported. For the eleven cases, 62 low risk contacts (overlap in shifts of nurses with cases < 72 h before onset of symptoms) were reported, resulting in a primary attack rate of 18% (95% CI 10.2–29.0). With exclusion of previously infected nurses we found 52 low risk contacts for ten of the eleven cases, resulting in an attack rate of 19% (95% CI 10.7–31.9).

Contact tracing for household and other social contacts was performed by the regional Public Health Service in the area of residence in eight of the nine nurses. Four out of seven household contacts (57%) and three out of six close contacts (50%) tested positive for SARS-CoV-2, resulting in an secondary attack rate of 54% (95% CI 29.1–76.8).

An outbreak management team was formed on December 29, 2020 when the Alpha variant was detected using sequencing. From that date, voluntary nasopharyngeal swabs were taken from all SARS-CoV-2-negative nurses working at the affected ward twice a week to detect asymptomatic cases. No additional cases were identified and the screening was ended in the beginning of January. Feasibility and deviations of the infection control measures were discussed with all cases. Three nurses reported to have developed symptoms during working hours. Short conversations in changing rooms without masks, short periods of lack of social distancing during work breaks and incorrect wearing of masks were mentioned as potential causes for workplace transmission. Masks were inadvertently touched during patient care and inappropriately removed. No additional private contacts outside of work were reported.

## Genomic epidemiology

On day 13 of the outbreak, sample NURSE-006-S1, collected from nurse 006, was sequenced during surveillance of randomly selected SARS-CoV-2 samples at the hospital and was found to be the Alpha variant. Consequently, all available outbreak samples with a cycle threshold (ct) value < 32 from that ward were sequenced subsequently and classified as lineage Alpha variant (Table 1). Amplification and sequencing were performed using the optimized Nanopore protocol and the ARTIC V3 amplicon sequencing protocol (https://artic.network). The resulting raw sequence data was analyzed using an in-house pipeline (https://github.com/RIVM-bioinformatics/TrueConsense).

**Table 1:** Dates and CT values of positive RT- PCRs at detection and on return to work, including serology results

Sample	Days into outbreak at day of detection	Ct value at day of detection	Day of return-to-work	Ct value at day of return-to-work	Day of serology results	Serology results ^$^ (Wantai total antibody score)
NURSE-001-S1	3	19	Day 23	Negative	Day 23	7.04
NURSE -002-S1	4	27	Day 23	31	Day 23	20.07
NURSE -003-S1	8	19	Day 23	33	Day 23	18.38
CAREGIVER-004-S1	7	21	–	–	–	–
CAREGIVER-005-S1*	8	*NA*	–	–	–	–
NURSE -006-S1	9	17	Day 26	32	*NA*	*NA*
NURSE -007-S2	11	31	Day 24	Negative	Day 24	20.58
NURSE -008-S1	12*	NA*	Day 29	29	Day 46	15.04
NURSE -009-S1#	10	34	Day 26	Negative	Day 26	19.15
NURSE -010-S1	15	20	Day 30	40	*NA*	*NA*
NURSE -011-S1	16	*NA*	Day 30	32	*NA*	*NA*

*NA* Not available= not applicable

*Not tested in hospital

^#^Not sequenced due to high Ct value

^$^Cut-offs for positivity were set at 1.1

**Fig. 2:**
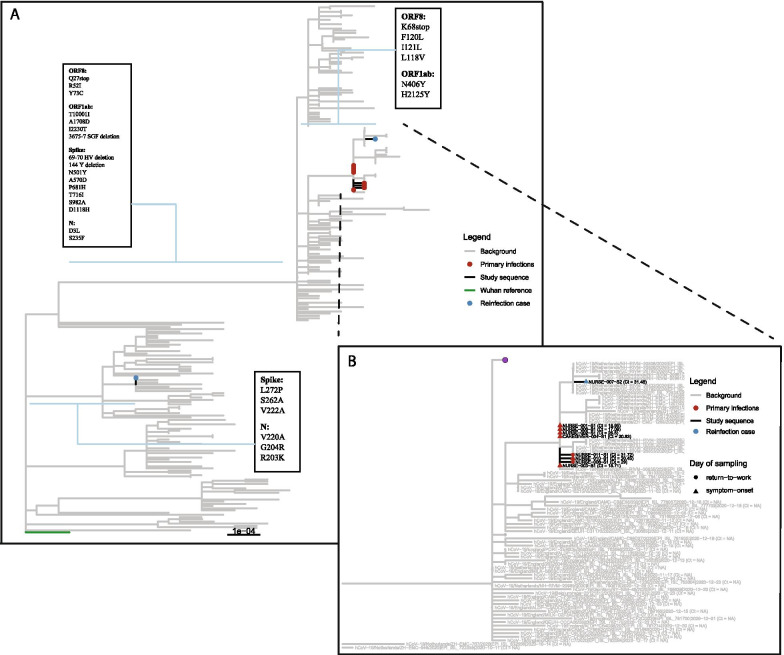
**a** Maximum-likelihood tree depicting sequenced samples and amino-acid differences between reinfection sequences. Red indicates outbreak samples. Blue circles reflect sequences derived from reinfection case. Boxes indicate the amino-acid changes relative to the Wuhan-1 reference genome along the indicated branches. Contemporaneous background sequences were derived from GISAID and are depicted as grey branches. **b** B.1.1.7 clade of **a** with focus on outbreak sequences. Red indicates outbreak samples. Purple dots represents a collection of collapsed branches of background sequences. When samples were unavailable at day-of-detection, samples collected at day of return-to-work were used where available. Contemporaneous background sequences were derived from GISAID and are depicted as grey branches

Eight out of nine sequences from outbreak samples showed the characteristics of transmission within the ward: a tight phylogenetic cluster closely related in both time (13 days between first and last positive test) and genomic diversity (either an identical sequence or 1 SNP). Study sequences were compared to contemporaneous sequences (derived from GISAID) and sequence NURSE-007-S2 was more similar to contemporaneous Alpha variants from the Netherlands (1 nucleotide difference) than to other sequences from the outbreak (Fig. 2b) to which it differed by 2 non-synonymous SNPs (Fig. 2b, ORF1a: H2125Y, ORF8: L118V). This would suggest that introduction from the community was a more plausible source of infection than hospital transmission. However, during the outbreak period, the national relative contribution of Alpha was estimated at less than 4% [4]. Moreover, this nurse had clear epidemiological links to other nurses at the ward. Nurse 007 had an overlapping shift on day 5 with a nurse that experienced COVID-19 related symptoms that day (Fig. 1, Nurse 003) and several overlapping shifts with nurses testing positive on day 15 and 16. Overall, the epidemiological data combined with phylogenetic analysis strongly suggests within-hospital transmission was the main route of transmission during this outbreak, but the source of transmission for NURSE-007-S2, the reinfection case, remains uncertain. Samples NURSE-007-S2 (collected day 10) and NURSE-007-S1 (collected 83 days prior to NURSE -007-S2) were both collected from nurse 007 and were unrelated (Fig. 2a, blue circles), thereby confirming a reinfection with an Alpha variant within three months after primary infection with lineage B.1.177, that was dominant in Europe since the summer of 2020 [1]. The amino acid differences between the two infections are listed in Fig. 2a, boxes 1–3.

## Discussion and conclusion

The SARS-CoV-2 Alpha variant was first detected in the United Kingdom in November 2020 and spread towards dominance due to its higher transmissibility in many countries including the Netherlands [1, 3]. Understanding transmission dynamics of differing variants among HCWs is important to ensure adequate containment and prevention measures are in place. Three key points were taken from this outbreak.

Firstly, a rise in SARS-CoV-2 cases at an orthopedic ward was confirmed to be an outbreak using genomic sequencing, with most genomes being identical or 1 SNP different. This outbreak with variant Alpha was controlled by adherence to existing guidelines, despite increased transmissibility of the variant illustrated by high attack rates to household members (57%%, compared to 16.6% previously estimated among 77 758 household members from 54 relevant studies) and close contacts (50%, compared to 5.9% among family and friends, 1.9% in the workplace, and 1.2% among casual close contacts) [5, 6]. This reiterates the importance of adherence to prevailing infection prevention methods to prevent transmission among HCWs. Interestingly, no high-risk contacts were reported by the 11 HCWs that tested positive. This could indicate an increased risk for transmission during low-risk contacts during an outbreak with the Alpha variant. It is more likely, however, that self-reported risk behavior was underestimated [2]. This is congruent with the fact that increased adherence to existing guidelines controlled the outbreak.

The outbreak management team was formed at day 13, directly after the results of sequencing reported the Alpha variant and universal testing for nurses was added to the containment strategy. Although the outbreak was controlled within a short period of time (16 days), three nurses developed symptoms during their shifts. Universal testing earlier into the outbreak, might have reduced the number of affected nurses and has been introduced as a routine policy in our hospital since this investigation [7]. Two of the cases in this outbreak were family members who were involved in the caregiving process. The family members did not leave the patient room and both family members and nurses wore masks when providing care for the patient in question. As the incorrect wearing of masks was presented by the nurses as a potential cause for transmission we suspect that the inadvertently touching of masks during patient care, and thereby contamination of the surroundings of the patient might explain nurse to family member transmission. It cannot explain why the patient itself was not affected. Other visitors were rarely present at the ward during this outbreak and were able to adhere to social distancing policy. We hypothesize that visitors were therefore less at risk to contract the infection. Secondly, while rare, reinfections with the Alpha variant can occur rapidly after primary infection with a preceding lineage such as B.1.177. Reinfection rates associated with the Alpha variant prevalence are similar to the rates of preceding lineages, but a reinfection within 90 days remains noteworthy [8]. Guidelines state that a positive PCR result within 90 days of initial symptom onset without new symptoms (as was the case in here) is unlikely to be the result of a reinfection, but rather represents persistent shedding of viral RNA [9]. This study highlights that viral sequencing is an essential tool to investigate new positive PCR results with 90 days of primary infection.

Lastly, although sequence analysis suggested that the reinfection case was unrelated to the outbreak based on the 2-nucleotide difference, the strong epidemiological links with cluster members, in combination with a low a priori risk of a community source (< 4% Alpha variant at the time), strongly suggests it is plausible that this case was in fact related to the outbreak. If the reinfection case was indeed related to the outbreak, the two mutations observed could reflect true evolutionary changes in the viral genome (the average rate is 2 substitutions per month) or, and perhaps more likely, could reflect some form of technical artefact, which have been discussed elsewhere [10]. This outbreak illustrates the complexity of transmission cluster inference for viruses with low genetic diversity such as SARS-CoV-2: identical sequences are not sufficient evidence for transmission, but sequences with a one or two nucleotide differences are equally not sufficient evidence to exclude relatedness. Weighing the genomic data in light of the epidemiological context is essential to draw appropriate conclusions about outbreaks like the one described here.

## Data Availability

All data generated or analysed during this study are included in this published article.

## Abbreviations

SARS-CoV-2: Severe acute respiratory syndrome coronavirus 2
HCW: Healthcare worker
SNP: Single Nucleotide Polymorphism
